# Insufficient evidence for interactive or animated graphics for communicating probability

**DOI:** 10.1093/jamia/ocae123

**Published:** 2024-06-21

**Authors:** Jessica S Ancker, Natalie C Benda, Brian J Zikmund-Fisher

**Affiliations:** Department of Biomedical Informatics, Vanderbilt University Medical Center, Nashville, TN 37209, United States; Columbia School of Nursing, New York, NY 10032, United States; Department of Health Behavior and Health Education, Department of Internal Medicine, and Center for Bioethics and Social Sciences in Medicine, University of Michigan, Ann Arbor, MI 48109, United States

**Keywords:** graphics, risk communication, interactive visualization, animated visualization, health behavior

## Abstract

**Objectives:**

We sought to analyze interactive visualizations and animations of health probability data (such as chances of disease or side effects) that have been studied in head-to-head comparisons with either static graphics or numerical communications.

**Materials and Methods:**

Secondary analysis of a large systematic review on ways to communicate numbers in health.

**Results:**

We group the research to show that 4 types of animated or interactive visualizations have been studied by multiple researchers: those that simulate experience of probabilistic events; those that demonstrate the randomness of those events; those that reduce information overload by directing attention sequentially to different items of information; and those that promote elaborative thinking. Overall, these 4 types of visualizations do not show strong evidence of improving comprehension, risk perception, or health behaviors over static graphics.

**Discussion:**

Evidence is not yet strong that interactivity or animation is more effective than static graphics for communicating probabilities in health. We discuss 2 possibilities: that the most effective visualizations haven’t been studied, and that the visualizations aren’t effective.

**Conclusion:**

Future studies should rigorously compare participant performance with novel interactive or animated visualizations against their performance with static visualizations. Such evidence would help determine whether health communicators should emphasize novel interactive visualizations or rely on older forms of visual communication, which may be accessible to broader audiences, including those with limited digital access.

## Introduction

Forty-three percent of United States adults and 90% of teens play video games.^1^ Video sites including TikTok and YouTube are ubiquitous sources of news and entertainment.^2^ Highly traditional news organizations such as the *New York Times* and the *Washington Post* now take pride in eye-catching interactive graphics to storyboard complex articles and illustrate quantitative ideas. Clearly, in the third decade of the 21st century, computer animations and computer-based interactivity are integral to the way many people learn and enjoy recreation.

Yet scholarly research on graphical risk communication has focused on 2 20th-century technologies, images that are printed on paper and images that are electronic but static and unmoving. Our systematic review on the effect of format on the communication of health-related numbers to lay audiences discovered more than 180 research studies that rigorously evaluated some sort of visualization, but only 24 of them included any sort of animated or interactive graphics.

In this perspective article, we describe 4 types of animations and interactive graphics that have been studied multiple times in the literature on communicating probabilities in health: visualizations that simulate experience, demonstrate randomness, direct attention, and promote elaborative thinking. We summarize the somewhat disappointing results of these studies and propose other areas for informatics research.

## Methodological background

This article is a commentary on some of the findings from our systematic review, which is being published fully elsewhere (Ancker et al. Scope, methods, and overview findings for the Making Numbers Meaningful evidence review of communicating probabilities in health: A systematic review. In press, *MDM Policy & Practice*. DOI: 10.1177/23814683241255334; Ancker et al. How point (single-probability) tasks are affected by probability format, part 1: a Making Numbers Meaningful systematic review. In press, *MDM Policy & Practice*. DOI: 10.1177/23814683241255333; Ancker JS, Benda NC, Sharma MM, et al. How point (single-probability) tasks are affected by probability format, part 1: a making numbers meaningful systematic review. *MDM Policy Pract*. 1. In press).^3^^,^^4^ In brief, in the review, we searched for studies that (1) compared 2 or more formats for communicating numbers in health, (2) enrolled lay (not medically trained) adults, and (3) assessed the effects of these formats using quantitative measures of affective, perceptual, cognitive, or behavioral outcomes. (The search approach, inclusion/exclusion criteria, instruments used for data extraction and risk of bias assessment, extracted data, and other tools from the review are reported in and freely available at our Open Science Framework project https://osf.io/rvxf2/.) The requirements for comparative assessments and quantitative outcomes excluded many high-quality human factors studies that applied exclusively qualitative methods, as well as data visualization studies using data not directly relevant to health. Nevertheless, the search produced 181 research studies assessing responses to graphics that communicated health probabilities, such as chances of disease, side effects, mortality, or other health events.

Of these, 24 employed animations or interactivity. We defined animations as graphics, videos, images, or slide shows that moved dynamically, with or without the participant having to do anything. We defined interactivity as the use of affordances that allowed participants to click, input information, draw graphics, or otherwise interact with the information. We found studies that examined animations, studies that employed interactivity, and studies that did both.

In reflecting on the findings of the systematic review, we were struck by how rare research into animation or interactivity was, and how few types of animation had been explored. When we conducted the review, we lumped all types of animations together and segmented the literature by the outcomes measured (eg, health behavior, risk perception, or recall). However, in examining the role of animations and interactive graphics, we propose that it might be helpful instead to analyze the types of visualizations studied. We here present a novel classification of animations and interactivity (graphics for simulating experience, for demonstrating randomness, for directing attention, and for promoting elaborative thinking) and provide theoretical background from cognitive psychology, education, and related fields about how each might be expected to exert its effects.

### Simulating experience: animation to create experiences of probabilistic events

Humans make some decisions based on experience and others on the basis of what we have read or been told. So, for example, we might estimate the likelihood of picking the Queen of Diamonds from a card deck on the basis on our experience of playing card games, whereas our estimate about the likelihood of getting into a car crash on our summer vacation road trip is probably based on reading news articles. Cognitive psychology evidence shows that judgments based on experience are quite different from, and in some ways better than, judgments based on description.^5^^,^^6^ Some researchers have attempted to apply this phenomenon to health communication by developing computer animations that provide a “simulated experience” of a probability (eg, a sequence of photos of people, 10% of whom have the condition). These studies were within scope of our review if they contrasted the simulated experience with some form of “described” probability (eg, “you have a 10% chance of getting this disease” or an icon array displaying 9 healthy people and 1 sick person).

**Figure 1.**
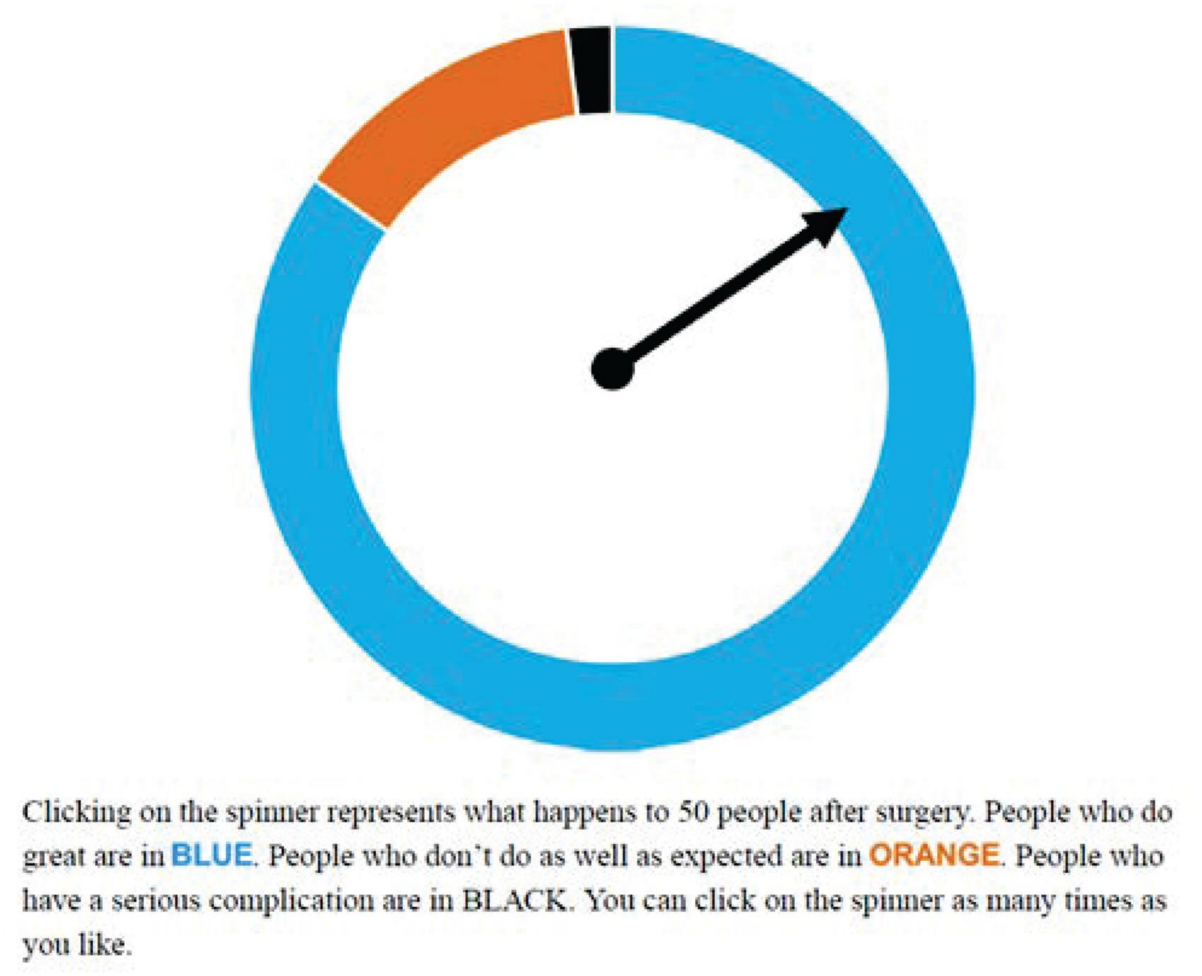
Interactive animation with original explanatory caption. Clicking on the image caused the pointer to spin around and come to rest at different points around the circle. Reproduced with permission from *Arthritis Care and Research* (Fraenkel et al^7^).

For example, Fraenkel and colleagues^7^^,^^8^ developed 2 types of experiential graphics. One was a series of images of people with and without the condition displayed one after another in a slide show, and the other was an interactive electronic spinner with a dial segmented to illustrate the proportions of people expected to experience different outcomes from a therapy (ie, improvement, no improvement, and adverse effects). Participants could click to spin the dial as many times as they wished (Figure 1). Ancker et al^9^ created a matrix in which participants clicked on squares to learn whether they concealed a person affected by the condition or a person not affected. Witteman et al’s icon arrays revealed 1 icon at a time but without the need to click.^10^

Simulated experience animations have not yet produced major beneficial effects on health communication. When compared to static graphics, the simulated experience animations did not improve knowledge,^7^^,^^8^ recall,^10^ or risk perceptions,^7^^,^^9^ and often did not change intended behavior.^7^^,^^8^^,^^10^ In addition, we did not find strong evidence that people liked simulated experience graphics better than static graphics.^9^ In experiments that tested the simulated experience animations against numbers without any graphics,^7^^,^^11^ some researchers found the animations produced better knowledge, ability to compute answers, and altered decisions or intended behaviors, but in these cases, because the control arms had no graphics, it seemed likely that at least some of the effect was simply due to the presence of graphics.

Some researchers used simulated experience through slideshows^11^ or icon arrays^12^^,^^13^ to tackle the problem of Bayes’ Theorem, specifically the challenge of showing the probability of disease with a positive screening test. Although these researchers were able to demonstrate some improvements with the interactive graphics, the fact that the comparison arms used numbers alone means it was not clear how much of the improvement was simply attributable to the graphics alone, rather than to the animation.

### Demonstrating randomness: animation to illustrate the randomness of probabilistic events

**Figure 2.**
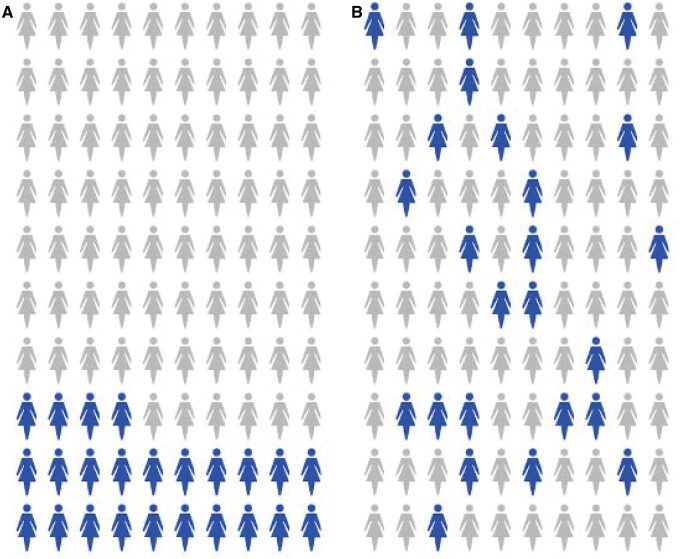
(A and B) Animations, with or without interactivity, designed to convey the uncertain and random nature of probability. Some studies used interactive features that allowed the participant to toggle between grouped (A) and randomized (B) versions of the array or to re-randomize the icons in the randomized one. Other studies used icon arrays in which the colored figures moved dynamically throughout the array or were revealed one at a time.

A key challenge in describing the probability of some health event is how to convey the stochastic nature of chance: we can predict what proportion of a large group of people will be affected, but not which people. In our review, we found several examples of dynamic icon arrays designed to demonstrate the randomness of a health probability (Figure 2). Witteman et al,^10^ Housten et al,^14^ Zikmund-Fisher et al,^15^ and Han et al^16^ used icon arrays in which the icons moved or were revealed sequentially at different positions throughout the matrix to emphasize the randomness of chance (in the first 3 of these studies, icons first appeared randomly and then settled in a group). Ancker et al invited participants to click on an icon array to re-sort the icons in random arrangements.^9^

Again, the studies we found did not suggest that animated randomness promoted health communication goals. When compared to static versions, the animated graphics did not alter perceived risk,^16^ improve the credibility of the information,^14^ improve ability to answer knowledge questions,^14^ improve ability to pick the largest probability,^15^ or alter intentions around health behaviors.^10^ One of these studies in fact showed that performance was worse with the animated icon arrays, and that people preferred the static ones.^15^

### Directing attention: animation to reduce information overload by directing attention to items of information in sequence

Information overload is a threat whenever we present complex information to patients. Animation or storyboarding could in theory address this threat by directing people’s attention to smaller chunks of information, which could be sequenced to help them reason through a problem or understand an argument. In our literature review, we found several examples of storyboarding animations or interactivity designed specifically to communicate quantitative information.

Housten and colleagues^14^ and Okan et al^17^ both used icon arrays with sequential reveals or highlighting to draw respondents’ attention to successive groups or subsets of icons. Neither found that the animations improved understanding (measured through having respondents do computations,^17^ answer knowledge questions, or identify the higher or lower option^14^) compared to static icon arrays, although Okan found that the animated versions were rated more positively by respondents.^17^

Sequential animations to direct attention have been used several times for Bayes’ Theorem communications about how the probability of disease is affected by a positive screening test result. Ottley created a storyboard in which sets of patients (disease-positive and -negative patients, positive and negative test results) were successively superimposed on an icon array.^18^ Tsai and colleagues used a similar set of superimposed sets on icon arrays, but additionally gave participants the ability to click the image to turn on and off the superimposed sets.^19^ The Ottley study found no advantage to the sequential animation over a static visual. Although the Tsai study demonstrated better performance with the sequential animation than with a static one, it was a tiny study of only 36 participants, all of whom were university students, limiting our confidence in generalizability or replicability.

### Promoting elaborative thinking: interaction to help process information more deeply

The educational literature on multimedia learning demonstrates that active interaction with information can promote elaborative thinking and deeper engagement with the information.^20^^,^^21^ Some health communication researchers have explored adding interactive exercises to help respondents process the quantitative health information they read. Natter and Berry invited participants to take a described numerical probability and use it to draw a bar chart, then compared performance with participants who merely read the probability.^22^ Mason et al contrasted a static bar chart and an interactive one in which the respondent was invited to adjust the height.^23^ Zikmund-Fisher et al^24^ invited respondents to use a website to create a graphic that illustrated the described probability.

Much of the interactive learning that has been tested to date did not have major benefits in terms of recall^23^ or satisfaction with the information.^22^ One study found an effect on perceived probability, but no accompanying effect on behavioral intention.^22^ Zikmund-Fisher and colleagues even found that the additional graph drawing exercise was associated with worse ability to select the best therapeutic option.^24^

Risk personalization and tailoring has also been employed to improve engagement through interactivity. Emmons et al^25^ and Harle et al^26^ used interactive risk calculators that allowed people to alter their modifiable risk factors to see the effect on the chance of developing colorectal cancer and diabetes, respectively. Compared to participants who read similar non-interactive information, the interactive groups did not have significantly more accurate risk perceptions^25^^,^^26^ or report being more satisfied with the information.^25^

## Discussion

Animation and interactivity in computer visualizations of probabilistic information have not shown strong evidence of promoting health communication goals such as altering risk perceptions, improving trust, or changing behaviors. There were actually examples of animation or interactivity degrading performance on comprehension-related tasks. Although there are a few counterexamples of studies with positive effects, the overall impression is of failure.

Is it simply that the research community has not rigorously assessed the types of sophisticated, immersive, interactive animations that would truly be more effective than static messages? We focused narrowly on the communication of numbers, not on the broader topics of health education and behavior change, which excluded innovations such as studies of the use of virtual reality for health education. We excluded studies that developed highly novel visualizations if they did not compare them quantitatively against more standard communication approaches, or if they did not present health data. We also recognize that there are many high-quality wellness and disease-management apps with sophisticated interactive features, but these did not appear in our review, again probably because they were not evaluated against comparable static information or appear in the peer-reviewed literature. Other sophisticated approaches to interactive visualization would not have been included because they lie outside of the health domain, including consumer apps, sports and fantasy-gaming sites, and video-gaming platforms.

Or do animations and interactivity genuinely lack effectiveness for risk communication? A paper co-authored by one of us declared a particular interactive graphic to be “cool but counterproductive.”^24^ Although there are plausible reasons to believe that animation and interactivity have potential benefits in health risk communication, the current evidence base does not provide much support for such claims. It is possible that because animation and interactivity are attention-grabbing, they can actually distract readers from the core task of risk communication, which is to evaluate the magnitude of the probability or ratio being visually displayed.

It is important to note that static visualizations, especially those that can be printed, are likely to be accessible to broader audiences of patients including those with limited broadband access^27^ or digital literacy.^28^ Thus, we should rely on evidence before replacing them with interactive or animated ones. At the moment, our message to anyone asking whether there is strong evidence to support using interactivity or animation for the communication of health probabilities is no—at least not yet.

## Data Availability

The data availability statement appears in the Methodological Background Section above, and reads: “The search approach, inclusion/exclusion criteria, instruments used for data extraction and risk of bias assessment, extracted data, and other tools from the review are reported in and freely available at our Open Science Framework project https://osf.io/rvxf2/.”
